# Effect of different drugs for controlling post-operative swelling after implant surgery among Indians

**DOI:** 10.6026/97320630019488

**Published:** 2023-04-30

**Authors:** Karthickraj Selladurai, Sahana Selvaganesh, Vishnu Priya Veeraraghavan, Thiyaneswaran Nesappan, Rajalakshmanan Eswaramoorthy

**Affiliations:** 1 Department of Implantology1, Saveetha Dental College and Hospitals, Saveetha Institute of Medical and Medical and Technical Sciences, Saveetha University, Chennai 600077, India; 2 Department of Biochemistry, Saveetha Dental College and Hospitals, Saveetha Institute of Medical and Medical and Technical Sciences, Saveetha University, Chennai 600077, India; 3 Department of Biomaterials, Centre of Molecular Medicine and Diagnostics (COMManD), Saveetha Dental College and Hospitals, Saveetha Institute of Medical and Medical and Technical Sciences, Saveetha University, Chennai 600077, India

**Keywords:** Management, inflammatory complications, pain, drugs

## Abstract

Pain and swelling are common complications associated with dental implant surgery. Forty five patients were included in this study (Group 1: Paracetamol+ amoxicillin (n=15), Group 2: Paracetamol+ Cold packs (n=15), Group 3: Paracetamol (n=15)). Post op drugs were given based on the group, and Pre and post-operative photographs were evaluated with Adobe photoshop software. The photographs were evaluated with Adobe Photoshop for Statistical analysis was done by repeated measures ANOVA. The first day post-surgery, there was increased swelling in group 1 with mean surface area of swelling of 47.6±2.1 mm2 and considerable decrease in group 2, 42.1±3.5 mm2. The surface area of swelling in this group was maintained in the same range till Day 7.

## Background:

Implantology is the growing field in dentistry that rehabilitates the oral cavity [1]. With the fast passed life and the necessity to get back to normalcy within a short span of time after surgery, the responsibility on the doctors increases, the lesser post-operative swelling and inflammation depends on various factors such as the operative surgeons skill, the post-operative medications taken by the patient and also the adjuvant therapies that are recommended to the patients. Many clinicians have thus emphasized the necessity for better pain, swelling and trismus control in patients who undergo implant placement [3]. Several methods of controlling the immediate inflammatory response associated with the implant surgery abound in the literature. These include different surgical closure techniques with or without incorporation of drained use of drugs such as analgesics, corticosteroids and antibiotics. Other reported modalities include physical therapeutic methods such as cryotherapy and laser application [4]. Therefore, it is of interest to document the effect of different drugs for controlling post-operative swelling after implant surgery.

## Materials and Methods:

This study was conducted at the department of implantology Saveetha Dental College. This study was approved by the ethical committee. Forty five patients have been selected who required implant placement on one quadrant. They were segregated into 3 groups 15 patients in each group.

Groups Factor

Group 1 Paracetamol +Amoxicillin

Group 2 Paracetamol Cold facial pack

Group 3 Paracetamol

**Figure 1:**
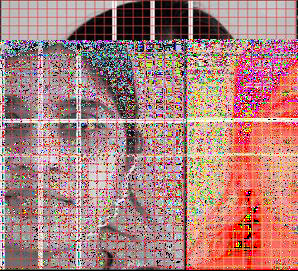
Grid placement to measure the volume of swelling

Pre op photographs of the patients were taken and they were given pre-emptive drugs respective of their group before 30 mins of the surgery. Three groups of treatment were provided post op and patients were separated into groups 1,2 and 3 (group 1 -paracetamol drug, group 2 - paracetamol +cold pack and group 3 -paracetamols) Duration of the surgery is also noted . Post op photographs were also taken to see the amount of swelling. Post op drugs are also given based on the groups. Photographs are all taken on day 1,day3 and day 7 and the photographs were evaluated with Adobe photoshop software, with formulation of grids on the photographs for all the 3 days for measuring the swelling.(Figure 1) The means were then calculated to assess the effectiveness for the particular group. The statistical analysis is done by repeated measures ANOVA in SPSS software.

## Inclusion criteria:

[1] Both Males and females of age between 18-60 years.

[2] Single or two implant placement in the same quadrant

[3] Minimal flap elevation.

## Exclusion criteria:

[1] Implant placement with augmentation procedures.

[2] Patient who was under antibiotic prophylaxis before surgical procedure

[3] Diabetes patient

## Results:

**Figure 2:**
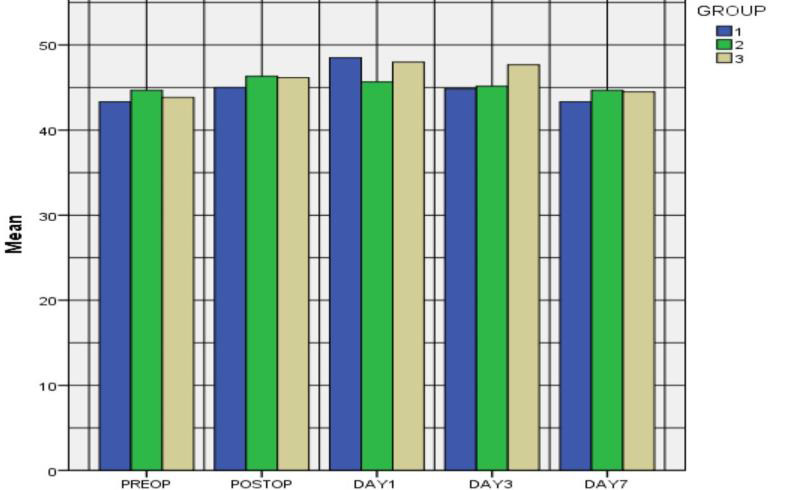
Graphical representation of different groups and volume of inflammation occurred during different time periods.

Patients in group 2 (cold facial pack - intermittent every fourth hourly on the day of surgery post op) had a much-reduced swelling in day1 and day3 with the mean and standard deviation of 42.1±3.5 mm2 and 40.3±2 mm2. Followed by the patients who had paracetamol + Amxicillin. Swelling reduced much slower rate in the group 3 (paracetamol) (Figure 2). This was compared with the volume analysis done with the pictorial restoration. There was no statistical significant difference between the three groups.

## Discussion:

Benefits attributed to local cold applications include, prevention of oedema by reducing the accumulation of fluid in body tissues, reduction in inflammation, slowing of metabolism, controlling haemorrhage, retarding bacterial growth, decrease in excitability of free nerve endings and peripheral nerve fibers with resultant increase in pain threshold, decrease in enzymatic activity, temporary decrease in spasticity, and a facilitation of muscle contraction. So this had better results in reducing the post inflammation at day1 to day 3 intervals. Scope for future research-More number of sample size and site specific must be taken into consideration. Duration of surgery should also be considered as criteria. Patient age and gender should also be considered.

##  Conclusion:

This article has presented the different modalities of management of pain and swelling in implant surgery. Post-operative swelling after implant placement is found to be more consideration for the patients nowadays, and the better post op medication and follow up instructions should give the patients a better option.

## Author contribution:

First author (S Karthickraj) performed the analysis and interpretation and wrote the manuscript. Second author (Sahana Selvaganesh) contributed to conception, data design, analysis, interpretation and critically revised the manuscript. Third author (N Thiyaneswaran) critically reviewed the manuscript. All the authors have discussed results and revised the manuscript.
